# Ceftolozane/tazobactam: Literature review of its activity on Taiwanese isolates before its launch in Taiwan (2012–2021)

**DOI:** 10.1016/j.heliyon.2024.e33114

**Published:** 2024-06-19

**Authors:** Chien-Ming Chao, Wen-Liang Yu

**Affiliations:** a Department of Intensive Care Medicine, Chi Mei Medical Center, Liouying, Tainan, 73657, Taiwan; b Department of Dental Laboratory Technology, Min-Hwei College of Health Care Management, Tainan, 73657, Taiwan; c Department of Intensive Care Medicine, Chi Mei Medical Center, Tainan City, Taiwan; d Department of Medicine, School of Medicine, College of Medicine, Taipei Medical University, Taipei City, Taiwan

**Keywords:** Ceftolozane, Ceftolozane/tazobactam, *Enterobacterales*, *Pseudomonas aeruginosa,* susceptibility, Taiwan

## Abstract

Ceftolozane, a novel cephalosporin, combined with tazobactam, a known β-lactamase inhibitor, shows robust antipseudomonal activity, although it doesn't cover carbapenemases. Our review of data from 2012 to 2021 in Taiwan highlights TOL/TAZ's in-vitro performance.

TOL/TAZ is most effective against *Pseudomonas aeruginosa* (91.3–94.4 % susceptible, with an MIC <4 μg/mL). It also demonstrates good activity against *Enterobacterales*, including *Escherichia coli* (88–94.3 % susceptible), *Klebsiella pneumoniae* (72.6–84.1 % susceptible), *Citrobacter koseri* (93.3 % susceptible), *Klebsiella oxytoca* (98.1–100 % susceptible), and *Proteus mirabilis* (100 % susceptible). However, its efficacy varies among species typically associated with chromosomally-mediated AmpC production, such as *Morganella morganii* (100 % susceptible), *Serratia marcescens* (81.3–90.0 % susceptible), *Enterobacter cloacae* species complex (76.6–76.7 % susceptible), *Klebsiella aerogenes* (66.7–89.6% susceptible), and *Citrobacter freundii* (60.0 % susceptible). For carbapenem-nonsusceptible isolates, TOL/TAZ is less effective against *K. pneumoniae* and *E. coli* (susceptibility <10 %) but remains useful for *P. aeruginosa* (susceptibility 81.3–91.8 %). In conclusion, TOL/TAZ shows potent activity against *P. aeruginosa* and carbapenem-susceptible *Enterobacterales* in Taiwan.

## 1 Introduction

Ceftolozane, a novel cephalosporin, has a structure resembling ceftazidime but with a modified side-chain at the 3-position of the cephem nucleus. This modification enhances its affinity for penicillin-binding proteins (PBPs), leading to strong antipseudomonal activity by blocking bacterial cell wall synthesis [1,2]. Notably, ceftolozane stands out from other β-lactams by being a potent inhibitor of PBP3 and having higher affinity for PBP1b and PBP1c [2]. Furthermore, ceftolozane is developed specifically to avoid efflux in *P. aeruginosa* and had high activity against isolates showing increased efflux expression [1]. Ceftolozane is susceptible to hydrolysis by ESBLs, AmpC β-lactamases, and carbapenemases [1]. The addition of tazobactam, a well-known β-lactamase inhibitor, broadens ceftolozane's effectiveness to include most ESBL and AmpC β-lactamase-producing organisms, while excluding those with carbapenemases like *Klebsiella pneumoniae* carbapenemase (KPC), some Ambler class D enzymes (such as OXA-48 and OXA-24), and metallo-β-lactamases (MBLs), such as IMP, NDM, and VIM [1,2]. Ceftolozane/tazobactam (TOL/TAZ), as a novel antipseudomonal β-lactam/β-lactamase inhibitor combination, demonstrates potent activity against multidrug-resistant *P. aeruginosa* and non-carbapenemase-producing *Enterobacterales* [1]. As TOL/TAZ was recently introduced to the Taiwanese market in 2022, we are conducting an efficacy review focused on Taiwanese *Enterobacterales* and *P. aeruginosa* isolates.

## 2 Current TOL/TAZ breakpoints

The Clinical and Laboratory Standards Institute (CLSI) has established breakpoints for *Enterobacterales* with regards to TOL/TAZ as follows: Susceptible, MIC ≤2/4 μg/mL; Intermediate, 4/4 μg/mL; and Resistant, >8/4 μg/mL [3]. For *P. aeruginosa*, the CLSI breakpoints for TOL/TAZ are: Susceptible, ≤4/4 μg/mL; Intermediate, 8/4 μg/mL; and Resistant, >16/4 μg/mL [3].

## 3 In-vitro activity globally

### 3.1 In Europe

TOL/TAZ showed strong efficacy against *Enterobacterales*, with a 93.5 % susceptibility rate when the MIC was at or below 2 μg/mL. Among these, ESBL producers without carbapenem resistance (non-CR) were even more susceptible (MIC_50/90_, 0.5/8 μg/mL) compared to carbapenem-resistant (CR) isolates with high MIC_50/90_ values (>32/>32 μg/mL) [4]. For *P. aeruginosa* isolates, TOL/TAZ demonstrated 92 % susceptibility, while other antibiotics like piperacillin/tazobactam, ceftazidime, cefepime, and meropenem had lower susceptibility rates ranging from 73 % to 79 %. Notably, for *P. aeruginosa* isolates resistant to the four mentioned drugs, TOL/TAZ still showed good activity with susceptibility rates as high as 70.4 % [4].

### 3.2 In the United States

*P. aeruginosa* exhibited a high susceptibility of 97 % to TOL/TAZ [5]. Among *P. aeruginosa* isolates that were not susceptible to meropenem, 88 % still remained susceptible to TOL/TAZ [5]. In the case of 145 *Escherichia coli* and *Klebsiella pneumoniae* respiratory isolates primarily carrying CTX-M genes, TOL/TAZ effectively inhibited 83 % of these isolates with an MIC of ≤2 μg/mL. It outperformed cefepime, which had a susceptibility rate of only 15 %, and piperacillin-tazobactam, with a 74 % susceptibility rate [6].

### 3.3 In the Asia-Pacific region

TOL/TAZ exhibited strong effectiveness with a 91 % susceptibility rate against healthcare-associated *P. aeruginosa* isolates [7]. It also demonstrated good in-vitro activity, especially against ESBL-phenotype *Enterobacterales* without carbapenem resistance, with MIC_50/90_ values of 0.5/16 μg/mL [7]. Moreover, it effectively targeted most *Enterobacterales* (susceptibility >80 %), but was less effective against *Enterobacter cloacae* species complex (76.6 % susceptibility) and carbapenem-nonsusceptible *P. aeruginosa* (72.8 % susceptibility) [8].

In China, the CHINET Program collected 3400 distinct Gram-negative clinical isolates from 45 medical centers in 2018. Susceptibility rates to TOL/TAZ were 89.6 % for *E. coli*, 58.1 % for *K. pneumoniae*, and 89.5 % for *P. aeruginosa*. Conversely, carbapenem-resistant isolates of *E. coli*, *K. pneumoniae*, and *P. aeruginosa* had significantly lower susceptibility rates to TOL/TAZ, measuring 0 %, 1.5 %, and 69.6 %, respectively [9].

## *4* *In-vitro* activity in Taiwan

### 4.1 Isolates from intensive care units (ICUs)

In a 2016 study of 300 clinical isolates from seven ICUs in Taiwan, TOL/TAZ demonstrated high susceptibility rates: 93 % for *P. aeruginosa*, 88 % for *E. coli*, and 80 % for *K. pneumoniae* [10]. Notably, carbapenemase-encoding genes were detected in four ertapenem nonsusceptible *K. pneumoniae* isolates (three with *bla*_KPC_ and one with *bla*_OXA-48-like_), but not in *E. coli* and *P. aeruginosa* isolates [10]. These findings suggest that TOL/TAZ may be a suitable choice for empirical treatment of infections caused by *P. aeruginosa*, *E. coli*, and *K. pneumoniae* in Taiwan. However, caution is advised in the case of *K. pneumoniae* due to the potential emergence of carbapenemase production in ICU settings.

### 4.2 Isolates producing ESBLs

In 2017, TOL/TAZ was less effective against ESBL-producing *K. pneumoniae* isolates compared to *E. coli* isolates in Taiwan's teaching hospitals. The presence of the carbapenem-resistant (CR) phenotype influenced susceptibility, increasing nonsusceptibility from 5.1 % to 18.2 % for *E. coli* when including CR isolates [11]. The nonsusceptibility rates of ESBL-producing *K. pneumoniae* to TOL/TAZ were 38.7 % for non-CR isolates and 70.7 % for CR isolates [11]. Therefore, it's crucial to exercise caution when interpreting TOL/TAZ susceptibility data for ESBL producers, particularly when studies involve CR isolates.

### 4.3 Isolates producing carbapenemases

In 16 Taiwanese hospitals from 2017 to 2020, carbapenem-nonsusceptible *E. coli* (*n* = 26) and *K. pneumoniae* (*n* = 175) isolates had low susceptibility rates to TOL/TAZ, at 7.7 % and 9.7 %, respectively [12]. *E. coli* also exhibited limited susceptibility to other β-lactams, except for imipenem and meropenem (both 76.9 %). Despite 40.3 % of these isolates carrying carbapenemase-associated genes, primarily *bla*_KPC_, this didn't explain the drop in TOL/TAZ susceptibility rates to less than 10 %. We presumed that the significant disparity between the prevalence of carbapenemase production and the rate of TOL/TAZ resistance is likely attributable to other enzymes (such as high-level ESBL or AmpC production) that overcomes tazobactam's inhibitory effect on the enzymes.

The susceptibility rate to TOL/TAZ was found to be 81.3–86.6 % for Taiwanese *P. aeruginosa* isolates that were not sensitive to imipenem/meropenem between 2017 and 2020 [13,14]. Additionally, 91.8 % of 73 imipenem-resistant *P. aeruginosa* isolates examined in a different investigation in 2021 were sensitive to TOL/TAZ [15]. These indicate that most of the Taiwanese isolates of *P. aeruginosa* that are resistant to carbapenems have non-carbapenemase resistance mechanisms.

## 5 Chronology of TOL/TAZ susceptibility in Taiwan

**Fig. 1:**
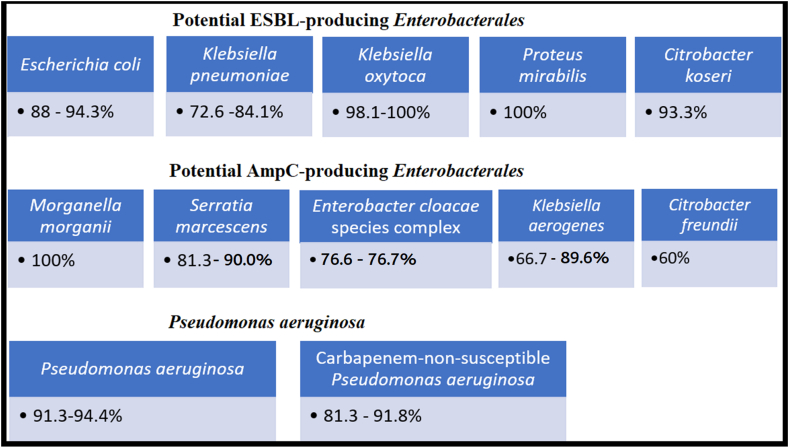
Activity of ceftolozane-tazobactam. A summary figure showing susceptibility rates of different bacterial groups to ceftolozane-tazobactam, along with *Enterobacterales* that may produce extended-spectrum-β-lactamase (ESBL) or AmpC β-lactamase; as well as *Pseudomonas aeruginosa,* all of which were collected from Taiwan (2012–2021) [8,[10], [11], [12], [13], [14], [15], [16]].

Based on the data mentioned above, it appears that Taiwan's multidrug-resistant *Enterobacterales* are becoming more resistant to TOL/TAZ activity. This is likely because of increased enzymatic hyperproduction and/or developing carbapenemase production. We tabulated data from several surveillance programs of the susceptibilities of Taiwanese *P. aeruginosa* and *Enterobacterales* of more diversified bacterial species and specimen sources for a more comprehensive evaluation that is not confined to specific bacterial species. We included information retrieved from papers in the PubMed system using the terms "Taiwan," "ceftolozane/tazobactam," and "susceptibility". We excluded those publications mentioned Taiwanese isolates in the global collections or Asia-Pacific programs but lacked detailed data pertient for Taiwan. The studies focusing on clinical outcomes of the patients treated with TOL/TAZ that lacked comprehensive susceptibility information were excluded. Overall, we included 7 studies that were accessible to evaluate the TOL/TAZ susceptibility over time from 2012 to 2021 [8,[10], [11], [12], [13], [14], [15]]. These studies showed how resistance mechanisms evolved and were impacted by the different proportions of ESBL, AmpC, or carbapenemase in the cohort isolates. Furthermore, we included official information from the internal surveillance program of Merck Sharp & Dohme Corp [16]. The information was submitted to the Taiwan Food and Drug Administration in order to obtain drug approval. The data from the eight studies described above were collected before the launch of TOL/TAZ into the Taiwanese market. Based on the historical data on TOL/TAZ susceptibility in Taiwan, it is likely that *K. pneumoniae* had a pre-existing resistance to TOL/TAZ because of species-specific variables or secondary harm from other broad-spectrum β-lactams, such as cephalosporins and carbapenems, that were employed there. There are several characteristics of the Taiwanese experiences as the following description (Table 1). A summary figure showing differences in TOL-TAZ activity between Taiwanese bacterial groups (Fig. 1).

**Table 1:** *In-vitro* susceptibility of *Enterobacterales* and *Pseudomonas aeruginosa* isolates collected in 2012–2021.

Organism	% Susceptibility (no. of isolates tested)									
	Antibiotic	2012–2013^a^	2014–2015^a^	2016^b^	2015–2016^c^	2017^d^	2017–2019^e^	2017–2020^f^	2018–2020^g^	2021^h^
References		Su et al. [16]	Su et al. [16]	Liao et al. [10]	Kuo et al. [8]	Jean et al. [11]	Lob et al. [13]	Lee et al. [12]	Liu et al. [14]	Karlowsky et al. [15]
Inclusion criteria		-a	-a	ICU, GNB	LRTI	BSI	ICU, LRTI	CnSE	CnSPA	CRPA
*Escherichia coli*	C/T	–	93.6 (125)	88 (100)	91.9 (247)	94.3 (686)	–	7.7 (26)	–	–
	IPM	–	100 (125)	99 (100)	97.7 (476)	–	–	76.9 (26)	–	–
	MEM	–	100 (125)	100 (100)	98.0 (247)	–	–	76.9 (26)	–	–
*Proteus mirabilis*	C/T	–	100 (30)	–	100 (45)	–	–	–	–	–
	IPM	–	33.3 (30)	–	53.7 (82)	–	–	–	–	–
	MEM	–	100 (30)	–	100 (45)	–	–	–	–	–
*Klebsiella oxytoca*	C/T	–	100 (30)	–	98.1 (52)	–	–	–	–	–
	IPM	–	96.7 (30)	–	100 (95)	–	–	–	–	–
	MEM	–	100 (30)	–	100 (52)	–	–	–	–	–
*Klebsiella pneumoniae*	C/T	–	75.2 (125)	80 (100)	81.9 (569)	84.1 (673)	72.6 (201)	9.7 (175)	–	–
	IPM	–	88.8 (125)	91 (100)	94.9 (1226)	–	83.6 (201)	27.4 (175)	–	–
	MEM	–	94.4 (125)	92 (100)	94.4 (569)	–	90.6 (201)	42.9 (175)	–	–
*Klebsiella aerogenes*	C/T	–	66.7 (15)	–	89.6 (67)	–	–	–	–	–
	IPM	–	60.0 (15)	–	95.1 (123)	–	–	–	–	–
	MEM	–	93.3 (15)	–	100 (67)	–	–	–	–	–
*Morganella morganii*	C/T	–	100 (15)	–	–	–	–	–	–	–
	IPM	–	0 (15)	–	–	–	–	–	–	–
	MEM	–	100 (15)	–	–	–	–	–	–	–
*Citrobacter koseri*	C/T	–	93.3 (15)	–	–	–	–	–	–	–
	IPM	–	100 (15)	–	–	–	–	–	–	–
	MEM	–	100 (15)	–	–	–	–	–	–	–
*Citrobacter freundii*	C/T	–	60.0 (15)	–	–	–	–	–	–	–
	IPM	–	73.3 (15)	–	–	–	–	–	–	–
	MEM	–	100 (15)	–	–	–	–	–	–	–
*Serratia marcescens*	C/T	–	81.3 (16)	–	90.0 (99)	–	–	–	–	–
	IPM	–	87.5 (16)	–	64.8 (199)	–	–	–	–	–
	MEM	–	93.8 (16)	–	97 (99)	–	–	–	–	–
*Enterobacter cloacae*	C/T	–	76.7 (60)	–	76.6 (124)	–	–	–	–	–
	IPM	–	80.0 (60)	–	94.8 (229)	–	–	–	–	–
	MEM	–	100 (60)	–	96.8 (124)	–	–	–	–	–
*Pseudomonas aeruginosa*	C/T	91.3 (23)	94.4 (126)	93 (100)	92.3 (765)	–	94.0 (267)	–	81.3 (150)	91.8 (73)
	IPM	21.7 (23)	65.9 (126)	66 (100)	78.8 (1574)	–	69.7 (267)	–	0 (150)	1.4 (73)
	MEM	21.7 (23)	79.4 (126)	77 (100)	77.9 (765)	–	8 (267)	–	33.3 (150)	27.4 (73)

Note. -, no available data; C/T, ceftolozane/tazobactam; IPM, imipenem; MEM, meropenem; ICU, intensive care unit; GNB, Gram-negative bacteria; LRTI, lower respiratory tract infection; BSI, bloodstream isolate; CnSE, carbapenem-nonsusceptible *Enterobacterales*; CnSPA, carbapenem-nonsusceptible *Pseudomonas aeruginosa*; Taiwan SMART, Surveillance of Multicenter Antimicrobial Resistance in Taiwan Program; Asian SMART, Study for Monitoring Antimicrobial Resistance Trends in Asian surveillance programme.

a Organisms were isolated from specimens of intra-abdomen (*n* = 223), respiratory tract (*n* = 153), urinary tract (*n* = 149), skin and soft tissue (*n* = 55), blood (*n* = 11) and others (*n* = 8) from 9 medical centers in Taiwan. The susceptible breakpoints of TOL/TAZ for *Enterobacterales* and *P. aeruginosa* were MIC of ≤2/4 μg/mL and ≤4/4 μg/mL respectively [16].

b Organisms were collected from the intensive care units of seven teaching hospitals in Taiwan (Taiwan SMART 2016) [10].

c Organisms were isolated from lower respiratory tract infections in 33 hospitals in the Asia-Pacific region, including 8 hospitals (1533 isolates) in Taiwan (Asian SMART 2015–2016) [8].

d Organisms were bloodstream isolates including community- and hospital-acquired isolates collected from 16 teaching hospitals across Taiwan (Taiwan SMART 2017) [11].

e Organisms were LRTI isolates from 37 ICUs in seven Asian countries, including 8 sites (914 isolates) in Taiwan (Asian SMART 2017–2019) [13].

f Organisms (MIC >1 μg/mL for imipenem, meropenem or doripenem, or > 0.5 μg/mL for eartapenem) were collected from 16 hospitals in Taiwan (Taiwan SMART 2017–2020) [12].

g Organisms (imipenem MIC >2 μg/mL) were collected from 16 hospitals in Taiwan (Taiwan SMART 2018–2020) [14].

h Organisms (imipenem MIC >8 μg/mL) were collected from 9 hospitals in Taiwan (Taiwan SMART 2021) [15].

### 5.1 Potential ESBL-producing *Enterobacterales*

Overall, TOL/TAZ was found to be highly active against putative ESBL-producers, such as *K. pneumoniae* (72.6–84.1 % susceptible), *Citrobacter koseri* (93.3 % susceptible), *K. oxytoca* (98.1–100 % susceptible), and *P. mirabilis* (100 % susceptible). Limited TOL/TAZ susceptibility data have been reported for *C. koseri, K. oxytoca,* and *P. mirabilis* in Taiwan.

### 5.2 Chromosoml AmpC-producing *Enterobacterales*

#### 5.2.1 Diverse activities among AmpC producers

TOL/TAZ has varied levels of activity versus chromosomally-mediated AmpC producers. *M. morganii*, for instance, exhibited 100 % susceptibility to TOL/TAZ. Then, the TOL/TAZ susceptibility of the *Serratia marcescens* and *E. cloacae* species complex was moderate (81.3 % and 76.7 %, respectively). The lowest rates of susceptibility to TOL/TAZ were found in *C. freundii* and *K. aerogenes*, with respective values of 60.0 % and 66.7 %. AmpC overexpression and species-specific factors are two possible resistance mechanisms of AmpC producers to TOL/TAZ, as discussed in the next sections (4.2.2 and 4.2.3).

#### 5.2.2 Species-specific factors

Tazobactam has species-specific activity against chromosomally-mediated AmpC β-lactamases, such as moderate activity against *M. morganii*, but exhibits less activities against *Enterobacter, Serratia,* and *Citrobacter* [17,18]. In the meantime, ceftozolane may offer protection against the *M. morganii* AmpC enzymes, either because of its higher affinity for the bacterial PBPs or because of its decreased affinity for these enzymes [1,2]. Additionally, there is a correlation between varying PBP levels and high or low β-lactam resistance in *S. marcescens* [19]. The differences in AmpC producers' susceptibility to TOL/TAZ could be partially explained by these species-specific ceftozolane-PBP interaction variables.

#### 5.2.3 Various levels of AmpC production

AmpC enzymes generated in sufficient quantities by *Enterobacter, Serratia*, and *C. freundii* would resist tazobactam. In 10–37 % of isolates of *Enterobacter* spp., *Serratia* spp., and *C. freundii*, hyperproduction of AmpC-producing resistance to broad-spectrum β-lactams was found [20,21]. The different induction levels of AmpC enzymes of these isolates interacting with tazobactam may potentially be related to the diverse TOL/TAZ activities, as these resistance mechanisms are similar to those of other β-lactam/β-lactamase inhibitors, such as piperacillin/tazobactam [22]. Furthermore, different isolate cohorts may have varied AmpC induction levels, which might explain the different TOL/TAZ susceptibility rates of 66.7–89.6 % within the same species of *K. aerogenes*. Further research is required as there are currently no data on the AmpC hyperproduction of *Enterobacterales* in Taiwan that confers their resistance to TOL/TAZ.

### 5.3 Persistent high susceptibility in *Pseudomonas aeruginosa*

*P. aeruginosa* exhibited susceptibility rates of 91.3–94.4 % to TOL/TAZ. In terms of susceptibility rates to both carbapenems, TOL/TAZ exhibited the best activity against *P. aeruginosa* when compared to imipenem (65.9–78.8 % susceptible) and meropenem (77–79.4 % susceptible) in general, as well as the lowest susceptibility rates of 21.7 % for isolates from 2012 to 2013 (Table 1). Even among the isolates with high rates of carbapenem resistance, the TOL/TAZ activity against *P. aeruginosa* is continuously strong (>90 % susceptible) in various Taiwanese surveillance investigations conducted between 2012 and 2021 (Table 1).

In other countries, *P. aeruginosa* strains' resistance to TOL/TAZ has been attributed mostly to the mutation-dependent overproduction of intrinsic β-lactamase AmpC [23,24]. For instance, AmpC overexpression is caused by a mutation in AmpC gene, and the cloned AmpC variants have much higher TOL/TAZ MICs [24]. It is worth noting to closely monitor the emergence of TOL/TAZ-resistant *P. aeruginosa* strains due to the overproduction of AmpC enzymes in Taiwan.

## 6 Discrepancy of susceptibility between *K. pneumoniae* and *E. coli*

Regarding ESBL-producing *E. coli* and *K. pneumoniae* blood isolates (including carbapenem-resistant isolates), nonsusceptible rates of TOL/TAZ in 2017 were 18.2 % and 70.7 %, respectively [11]. The difference in TOL/TAZ susceptibility between *K. pneumoniae* and *E. coli* isolates suggested that the former had a greater prevalence of carbapenemase production, had a different β-lactamase gene, or had enzymatic hyperproduction. These findings were consistent with reports from the Asia-Pacific region [8]. Tazobactam may enhance or preserve the activity of ceftozolane by avoiding hydrolysis by ESBLs or AmpC β-lactamases, but not carbapenemases [1,2]. Therefore, resistance to TOL/TAZ often came from the carbapenem-resistant isolates due to the production of carbapenemases.

## 7 Evolution of carbapenemase genes in *K. pneumoniae* in Taiwan

The endemicity of carbapenemases has changed from imipenemase (IMP)-8, New Delhi metallo-β-lactamase (NDM)-1, and Verona integron-encoded metallo-β-lactamase (VIM)-1 to the most common *K. pneumoniae* carbapenemases (KPC)-2 and rapidly emerging oxacillinase (OXA)-48 in Taiwan during the period of 1998–2019 [25]. There are two steps to approach this issue**.** On one hand, rapid diagnosis of carbapenemase-producing *Enterobacterales* is important to make sure adequate treatment while using TOL/TAZ [26]. A recent study of carbapenem-nonsusceptible *Enterobacterales* collected in Taiwan revealed the proportion of carbapenemase production increased from 36.1 % in 2017 to 43.6 % in 2020. The most common carbapenemase gene was *bla*_KPC_ (79.3 %), followed by *bla*_OXA-48-like_ (13.8 %), *bla*_NDM_ (4.6 %), and *bla*_VIM_ (4.6 %) [12]. Carbapenemase genes were more frequently detected in *K. pneumoniae* isolates (47.4 %, 83/175) than in *E. coli* isolates (15.4 %, 4/26); nonetheless, the overall activity of TOL/TAZ was poor against carbapenem-nonsusceptible *K. pneumoniae* (9.7 % susceptible) and *E. coli* (7.7 % susceptible) isolates, with or without carbapenemase genes [12]. On the other hand, these data suggest that other mechanisms than carbapenemases additionally contributed to the TOL/TAZ resistance. Therefore, continuous surveillance for more clinical isolates from the latest periods and molecular characterization of the non-carbapenemase-producing carbapenem-resistant *Enterobacterales* should be further conducted to inform the clinical role of TOL/TAZ in such resistant isolates.

## 8 Low prevalence of carbapenemase genes in *P. aeruginosa* in Taiwan

Ceftozolane exhibits strong anti-*P. aeruginosa* activity; nevertheless, these isolates possess diverse and multifaceted mechanisms that confer resistance to imipenem, meropenem, PIP/TAZ, ceftazidime, and cefepime. Cefotozalane could evade a number of the *P. aeruginosa* resistance mechanisms, including efflux pumps, porin defects, and modification of PBPs, but it is inactive against isolates carrying the carbapenemases [1]. Thus, TOL/TAZ has often exhibited good effectiveness against *P. aeruginosa* isolates that are resistant to imipenem or meropenem in Taiwan, as these isolates frequently do not produce carbapenemases [9]. In fact, only 2.2 % (13/600) of the *P. aeruginosa* isolates collected between 2015 and 2021 possessed carbapenemase genes [15]. Furthermore, the outer membrane protein OprD is a preferred channel of entry for carbapenems in *P. aeruginosa* [27]. Nucleotide substitution or deletion of *opr*D gene is a key carbapenem resistance mechanism in *P. aeruginosa* isolates from Taiwan. This mutation was found in 95.2 % of these isolates [28].

Nonetheless, the Asia-Pacific region's carbapenem-resistant *P. aeruginosa* exhibits notable regional variations in the synthesis of carbapenemase. Particularly in India, *bla*_VIM_ (29.0 %) and *bla*_NDM_ (24.9 %) were the most prevalent carbapenemase genes [29]. Rarely in Taiwan, only 13 (2.2 %) of 600 *P. aeruginosa* isolates harboring carbapenemase genes, including 10 isolates with an MBL (six carrying VIM, one carrying IMP, and three co-carrying VIM and KPC) as well as three isolates with a KPC [15]. The presence of carbapenemase production may decrease TOL/TAZ's efficacy against *P. aeruginosa* that is not susceptible to carbapenems. For this reason, accurate antibiotic prescription depends on ongoing surveillance in Taiwan for *P. aeruginosa* carbapenemase production.

## 9 Conclusion

TOL/TAZ is highly active against a wide range of Gram-negative pathogens, including *Enterobacterales* and *P. aeruginosa*, which are primarily isolated from intra-abdominal, respiratory, and urinary specimens in Taiwan. With the exception of carbapenemases, the majority of *Enterobacterales* isolates that produce ESBLs or AmpC β-lactamases are vulnerable to TOL/TAZ. Additionally, TOL/TAZ continues to be effective against isolates of *P. aeruginosa* that are resistant to carbapenems, which do not often generate carbapenemases in Taiwan at this time. Nevertheless, when tested against *Enterobacterales* that produce carbapenemase, TOL/TAZ showed limited in-vitro performance. To understand the TOL/TAZ resistance mechanisms, it is necessary to investigate the enzymatic hyperproduction for *Enterobacterales*, especially *E. cloacae* species complex, *C. freundii*, and *K. aerogenes*. For accurate antibiotic prescription in Taiwan, it is crucial to continuously monitor for possible AmpC overproduction and carbapenemase production in *Enterobacterale*s and *P. aeruginosa*.

Clinicians should know that different TOL/TAZ susceptibility testing methods used in different studies might have a potential impact on activity results. In addition to conventional and time-consuming culture methods, the use of rapid multiplex PCR assay allowing early detection of carbapenemase genes (such as KPC, OXA-48, NDM, VIM, and IMP) can exhibit positive impact on administering appropriate antibiotics earlier and avoiding unnecessary prescriptions [30,31]. For instance, TOL/TAZ is suitable for *P. aeruginosa* that is resistant to carbapenem but lacks a carbapenemase gene, while other molecules such as ceftazidime-avibactam may be of interest to target isolates expressing either KPC or OXA-48 carbapenemase [32,33].

## Ethical statement

Ethical approval was not required. The study did not obtain any sensitive information and demographic data of the patients. No discernible information could be traced back to a patient source.

## Funding statement

Not declared by all authors.

## Data availability statement

No datastes were generated for the research described in the review article.

## CRediT authorship contribution statement

**Chien-Ming Chao:** Writing – original draft, Methodology, Data curation. **Wen-Liang Yu:** Writing – review & editing, Validation, Supervision, Conceptualization.

## Declaration of competing interest

The authors declare that they have no known competing financial interests or personal relationships that could have appeared to influence the work reported in this paper.
